# Development and Study of Ezzence: A Modular Scent Wearable to Improve Wellbeing in Home Sleep Environments

**DOI:** 10.3389/fpsyg.2022.791768

**Published:** 2022-03-17

**Authors:** Judith Amores, Mae Dotan, Pattie Maes

**Affiliations:** ^1^MIT Media Lab, Cambridge, MA, United States; ^2^Massachusetts General Hospital, Harvard Medical School, Boston, MA, United States

**Keywords:** wellbeing (I31), wearables, sleep, odor, olfaction, olfactory interfaces, human-computer interaction

## Abstract

Ezzence is the first smartphone-controlled olfactometer designed for both day and night conditions. We discuss the design and technical implementation of Ezzence and report on a study to evaluate the feasibility of using the device in home-based sleep environments. The study results (*N* = 40) show that participants were satisfied with the device and found it easy to use. Furthermore, participants reported a significant improvement in sleep quality when using the device with scent in comparison to the control condition (*p* = 0.003), as well as better mood the following morning (*p* = 0.038) and shorter time to sleep onset (*p* = 0.008). The device is integrated with a wearable EEG and real-time sleep staging algorithm to release scent during specific sleep stages (N1, N2, N3, and REM), which is important for certain use cases (e.g., to study the effect of scent on REM dreams, or to improve memory consolidation with a re-exposure of scent during N2 and N3). Ezzence can be used for several applications, including those that require scent triggered day and night. They include targeted memory reactivation, longitudinal health treatments, therapy, and mental or physical exercises. Finally, this article proposes an interaction framework to understand relationships between scents and environments based on proxemic dimensions and passive or active interactions during sleep.

## 1. Introduction

An estimated 50–70 million people in the United States of America suffer from sleep disorders (on Sleep Disorders Research, 2003). Sleep insufficiency and poor sleep quality are correlated with an increased risk of anxiety, diabetes, heart disease, stroke, obesity, and high blood pressure (Liu et al., 2016). Approximately 40% of people that look for medical assistance concerning sleep problems have a psychiatric disorder (Ford and Kamerow, 1989), and more than 70% of depressed patients have insomnia symptoms (Nutt et al., 2008). Insomnia, poor sleep quality, and insufficient sleep is a growing problem in society and is becoming more worrisome due to the COVID-19 pandemic. Poor sleep has been reflected in the increased number of sleeping aids bought globally and rising interest in non-pharmacological sleep interventions (Intelligence, 2018). This article investigates the feasibility of developing scent technologies to improve wellbeing and sleep in naturalistic environments, such as home-based studies. We focus on the use of scent as an intervention during sleep due to the olfactory sense's unique anatomical structure and its privileged connection to memory and the emotional part of the brain, which is especially relevant to develop applications to improve sleep quality and wellbeing. Unlike other sensory modalities, many types of olfactory stimuli can be presented during sleep without awakening (Carskadon and Herz, 2004), as well as during wake time without distracting users from their primary activity, thereby offering novel opportunities for interfaces and applications that extend from wake to sleep time. This research wished to assess the use of scent as an implicit, less conscious stimulus during sleep, that nevertheless has the ability to influence the person's cognition and sleep quality.

## 2. A Framework for Olfactory Interactions During Sleep

Human-computer interaction (HCI) has traditionally focused on designing technologies and studying how humans interact with them during wakefulness. However, we almost spend a third of our lives sleeping. Still, most of the technology developed for sleep applications focuses on sleep tracking rather than real-time interventions using non-traditional stimuli such as scent. In comparison to visual, haptic, and audio technologies, olfaction has been less explored in HCI. Human-computer interactions typically require a conscious, explicit input from the user to the system (e.g., typing on a keyboard or touching a screen), and typically the output from the computer to the user assumes that the person is awake (e.g., graphical user interfaces). In contrast, this article proposes computer interventions whose output changes based on minimal or no human input when the person is sleeping or awake. In particular, we explore the process and challenges of developing technology that uses scent as a stimulus in different naturalistic environments with the goal to improving wellbeing during day and night.

Olfactory feedback is an exciting yet to be exploited medium for improving sleep quality and interacting with a sleeping individual without disturbing them. This article proposes an interaction framework to understand relationships between scents for liminal day-night interactions and environments. In the following paragraphs we categorize Olfactory Interfaces (OIs) based on proxemic dimensions (which we will explain in the following section) and passive or active interactions.

### 2.1. Scent and the Environment: Olfactory Proxemics

**Figure 1:**
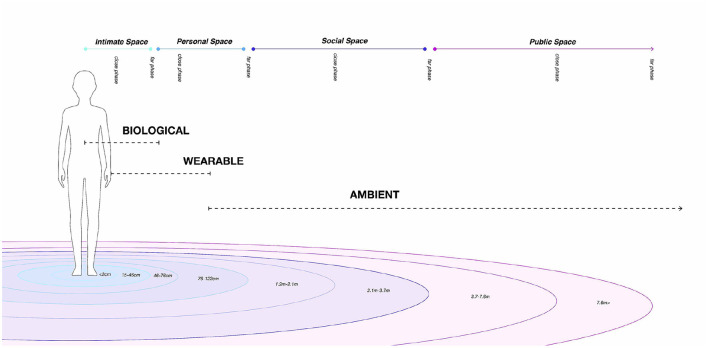
*Olfactory* proxemics. **Ambient**: generated in the environment by a source co-located nearby or far away from the user. The distance can range from a personal space to social space or public space (e.g., home diffusers). **Wearable**: worn by the user in a personal space (e.g., Ezzence wearable). **Biological**: generated in or on the user in an intimate space (e.g., chemosignals, scent hallucinations, synthetic scents).

Maggioni et al. (2020) described four key features around scent stimuli (i.e., chemical, emotional, spatial, and temporal) and proposed a “*Smell Space”* to use these features in smell-based interactions. In this article, we focus on the spatial features of smell. OIs are fundamentally different from other user interfaces, as scents can linger and disperse in space. Their intensity can very quickly change over time, and they can accumulate or disappear depending on the airflow and dimensions of the area. Like proxemics, scent can be classified based on its presence in a physical environment. Hall (1966) defines proxemics as “*the interrelated observations and theories of humans' use of space as a specialized elaboration of culture”* and classified the social interaction in a physical environment in four distinct zones: (1) intimate space, (2) personal space, (3) social space, and (4) public space. The framework we present expands on his work and applies it to the context of odor delivery and categorizes passive/active scent interactions with computers as Biological, Wearable, and Ambient based on the distance between the origin of the scent-delivery device and the user (as depicted in Figure 1).

### 2.2. Ambient OIs

Ambient scent technology can range from stationary olfactometers that are located at a personal distance, home diffusers that are placed at a social distance to large-scale, architectural scent delivery in public spaces (as described in Figure 1). These devices can also be co-located and allow remote synchronization of scent between users over distance. Ambient olfactory interaction can be understood as interaction with ambiently diffused scents (i.e., when a scent is occupying the entire space around, or nearby the user, e.g., examples of this term can be seen in the work by Bodnar et al., 2004; Brewster et al., 2006; Warnock et al., 2013).

#### 2.2.1. Olfactometers Used in Traditional Scientific Research

**Figure 2:**
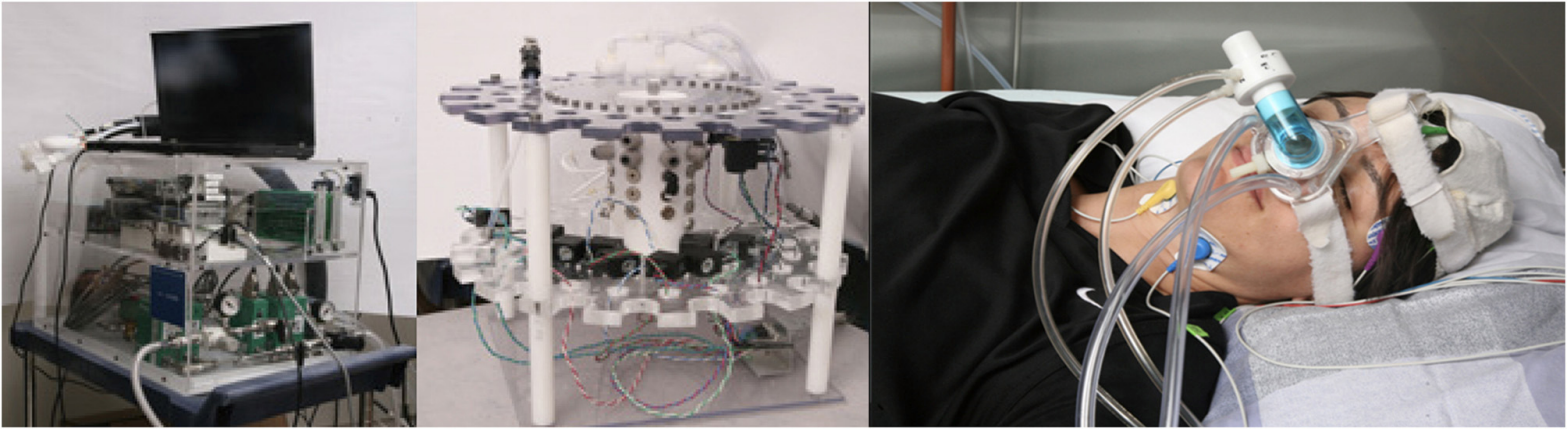
The images depict a typical sleep study using olfactory stimulation. The experiment is conducted in a laboratory with a nasal mask attached to an olfactometer that releases various scents stored in the bottles. Images by (Arzi et al., 2014) (CC-BY).

Olfactometers (or stationary computer-controlled scent delivery machines with nasal cannulas) are among the most widely used instruments for scent delivery in scientific studies. They are a standardized way to study olfaction and are widely referred to as “*olfactometer”* throughout the literature in Psychology, Neuroscience, Psychiatry, and clinical research (e.g., Carskadon and Herz, 2004; Rasch B. et al., 2007; Stuck et al., 2007). However, as per “Oxford Languages,” an olfactometer is “an instrument for measuring the intensity of an odor or the sensitivity of someone or something to an odor.” Thus, in other fields like Human-Computer Interaction or Electrical Engineering, it is often referred to simply as a scent-delivery device, olfactory interface, or olfactory display. These devices allow for precise administration of odor stimuli and generate a minimum delay between activation and arrival of the odor to the user. Current scent delivery systems for night-time studies consist of nasal masks/cannulas that are connected to large olfactometers via long Teflon tubes (see Figure 2).

The scent release device is usually placed in an adjacent room to avoid awakening the user due to the noises generated by the pumps. The technical expert sets up the device and controls the odor's duration and airflow through a computer connected to the device. There are several olfactometers on the market that vary from 3 to over 20 kg, the smallest found with four scent release weighs 4.6 kg and measures 31 L × 20 W × 15 H [cm] (see Scientific, 2019). Some research laboratories choose to build their olfactometers since the devices on the market can be costly (up to 100,000 USD, Lorig et al., 1999), but even the cost of custom ones can surpass 1,000 or 5,000 USD to build (e.g., Lundström et al., 2010; Lowen et al., 2017) and they typically exceed the dimensions of commercial systems. With the aim of creating an accurate olfactometer with smaller size and weight, Risso et al. (2018) created and tested an olfactory device that weighs ≈2 kg and is 50 L × 40 W × 40 H [cm]. As an alternative, some experimenters do not use olfactometers but instead hold bottles/vials of scent with their hands and intermittently present it to the participant while carefully controlling the exposure time and proximity to the nose to prevent habituation (see Goel et al., 2005).

Although the ultimate goal of these olfactometers is to provide scent delivery at an intimate or personal space, these scent delivery devices can not be worn and have to be placed on the side of the user due to their size. Therefore, we categorize them as “*Ambient*.”

#### 2.2.2. Ambient Scent Technologies in HCI

Some of the most recent advances in scent delivery systems have taken place in the field of HCI. Researchers have looked into designing olfactory technologies for a wide range of day-time applications. These devices tend to be more user friendly, smaller, and cheaper. For example, the prototypes developed by Herrera and McMahan (2014) or Dmitrenko et al. (2017) cost <1,000 USD, which is significantly less than the olfactometers built for traditional science studies. The majority of these scent delivery technologies are stationary, but a few are wearable. Kaye (2001) was one of the pioneers in using aromatic outputs in HCI and developed an automated scent-release mechanism, which facilitated the more objective study of scents for interactive applications. More recently, HCI researchers and product developers have focused on enabling scent to become part of digital communications (e.g., Strong and Gaver, 1996; Choi et al., 2011, 2013; Scentee, 2013; Aromajoin, 2016). Most systems use off the shelf aromas in their prototypes, focusing research efforts on the device itself. For example, the Ophone Ophone (2014) was a commercial product with 32 unique scents that could be combined to create tags for a message or a photograph to be sent through the Internet and reproduced on the other side. A similar idea was Scentee (2013), which emitted a fragrance when a notification is received on the user's smartphone. Some HCI researchers like Ranasinghe et al. (2011) explored the use of scent for digital communication, enabling the sharing of scent over the Internet. Dephemeral^1^, explored the future of teleporting flavors and scents through screens in a science-fiction video. Other researchers like Maggioni et al. (2018) have explored the use of olfactory notifications into a messaging application to improve users' confidence and performance in identifying the urgency level of a message. These types of notifications have also been researched for in-car user interfaces by Dmitrenko et al. (2019). Dmitrenko et al. (2016) proposed a three-dimensional framework to compare scent-delivery devices based on the distance, volume, and speed of scent-delivery to guide the design of in-car olfactory interfaces. Scent notifications were perceived as more comfortable, less distracting, and more helpful than visual notifications, which resulted in fewer driving mistakes (Dmitrenko et al., 2018). Related research by Dmitrenko et al. (2020) has also shown that scent of rose and peppermint might be able to calm drivers.

Some researchers have also explored the use of scent in games, like the work by Nakamoto et al. (2008) that lets users “cook” a virtual dish. Others have explored the release of scent though form; Clayodor by Kao et al. (2015) explores the mapping of a malleable tangible interface with scent. Fragrance has also been used to convey data in the work by Patnaik et al. (2018) and Batch et al. (2020).

The devices aforementioned are categorized in our framework as *Ambient* since they are often placed near the user, in a personal, social, or public space (as shown in Figure 1). An interesting approach that can be used to move the flow of scent across spaces (e.g., from the social space to personal and even intimate space), is the work proposed by Hasegawa et al. (2018). In their manuscript, they redirect the flow of scent by producing an electronically steerable ultrasound field. This technique has been used in the past to generate mid-air haptic sensations and might be useful to remove odors in the environment and to better control the space between the scent delivery device and the user. Finally, in the context of scents in digital art, Lai (2015) explored the role of an olfactory interface in an art museum to engage visitors around the artwork area.

#### 2.2.3. Large-Scale Scent Delivery for Urban Planning and Architecture

*Ambient* scent has been widely used throughout history in sacred places in many different religions. Burning incense is still used as an aid in prayer and spiritual practices for meditation and rituals in churches and temples, and probably, after all this use, some of these buildings already have the scent of incense impregnated in their construction materials.

Award-winning architect Peter Zumthor said once that “*Architecture is not about form, it is about many other things. The light and the use, and the structure, and the shadow, the smell, and so on.”* In his Bruder Klaus Chapel, layers of concrete were poured on top of a wooden frame that was later set on fire to create the internal structure of the chapel and left behind the scent of the burnt wood reminiscent of the initially formed space.

Beyond architecture, scent-delivery can also be used at an urban scale, although it has not been widely explored. Xiao et al. (2020) studied and proposed a framework around the perceptual process of smellscape perceptions. McLean (2019) creates olfactory landscapes and maps the scents of a city to render with different types of visualizations. Sissel Tolaas, Maki Ueda, and Peter de Cupere have also mapped odors from cities and different areas. Other artists such as Sam Bompas and Harry Parr have explored scent-delivery in large-scale environments and created multi-sensory fireworks, orange-flavored bubbles, and banana-flavored confetti. More recently, Spence (2021) investigated the role of ambient scent in passenger transportation and brought up very relevant areas of exploration such as air pollution, branding, marketing campaigns in the Underground and various forms of public transport, as well as scented terminals and stations.

### 2.3. Wearable OI

Scent delivery technologies primarily consist of stationary devices that are not designed to be worn. The research on wearable scent delivery technologies is still under-explored and mainly focused on ambient scent delivery rather than personal. There have, however, been a few exceptions described in the following paragraphs.

#### 2.3.1. Head-Mounted

The history of multi-sensory immersive scent research can be first dated to scents being first released during the viewing of a film, so that the viewer could associate certain scents with scenes of the movie (Smell-O-Vision^2^). In 1962, Morton Heilig patented Sensorama, which could be considered as the first virtual reality system. It was an immersive device, including stereo sound, scent, and tactile stimulation to provide a multi-sensory experience. Since then, researchers and designers have studied the effects of scent on VR experiences (e.g., Flavián et al., 2021) and developed head-mounted displays with wearable scent delivery like the work by Yamada et al. (2006) that uses scent in the outdoor environment, or Choi and Cheok (2015) with Sound Perfume, a pair of 3D-printed glasses embedded with a heating module for releasing the scent at the end of the frames. Other artists like Simun (2014) designed a low-tech artistic piece that is worn around the nose, held like glasses. Narumi et al. (2011) presented a head-mounted display to augment flavors and taste using edible markers for augmented reality. Other head-mounted scent delivery devices have been created, for example, the work by Hashimoto and Nakamoto (2016) and more recently Brooks et al. (2020) used trigeminal odors in VR to simulate temperature illusions (e.g., using eucalyptus for cooling and the chili pepper compound for warmth). Trigeminal odors activate the trigeminal nerve, a sensory nerve responsible for temperature sensations, tactile, pressure, and pain in the nose, eyes, and mouth. There are certain types of odors, such as menthol, Pyridine, peppermint, or capsaicin based fragrances that activate the trigeminal nerve and produce a cold or hot sensation and can cause arousals (e.g., see the work by Badia et al., 1990 or Carskadon and Herz, 2004). Finally, researchers like Ranasinghe et al. (2018) have also combined olfactory and haptic (thermal and wind) stimuli with physiological and subjective measures and proved that these types of VR experiences improve the sense of presence compared to traditional audio-visual feedback.

#### 2.3.2. On-Face OI

The social aspects of wearability are especially relevant when designing devices that are worn on the face. The social impact of non-traditional form factors such as on-skin interfaces located at the collarbone, ears, back of the neck, arms, forearms, and hands have been explored in the past by You et al. (2019). Researchers have developed various interactive technologies and sensors that are attached or worn on the face, such as head-mounted displays (HMD), wearable electroencephalograms (EEG), or nose interfaces for nostril temperature recording (see Kodama et al., 2018). Through the perspective of beauty technology, Kao et al. (2016) designed on-face dynamic color-changing eye shadow to create interactive body decorations. Limited research in the area of on-face olfactory interfaces has been done to date; Wang et al. (2020) describes “On-Face Olfactory Interfaces,” a series of form factors for olfactory wearables that are lightweight and can be attached to the skin or to glasses and piercings. The article also compares the user's perception and usability of these devices when pairs of participants interact at a close personal distance.

#### 2.3.3. Accessories and Fashion

Smart textiles can incorporate a variety of sensors and actuators; for example, Kan et al. (2015) uses thermochromic dye with conductive thread to reveal or make disappear words on a piece of textile. The t-shirt also has an embedded vibration motor on the neck that is activated when two people are nearby. Other smart textiles that respond when a person is approaching include the “Smoke Dress” by artists (Wipprecht and Casas, 2012) that releases a cloud of smoke. Light and temperature might also trigger changes. For example, “Climate Dress” has an array of LEDs that light up depending on the CO2 levels in the air (Design, 2009). Scientists like Goncalves et al. (2019) have explored the use of scent in textiles and have modified cotton fabric so that in contact with sweat, it releases β-citronellol, a fragrance obtained from the leaves of lemongrass that is commonly used as a mosquito repellent. Some artists have explored the use of scent in fashion such as Tillotson and Andre (2005) or ICT Scent Collar by Tortell et al. (2007), a scent delivery device in the form factor of a collar or bib to use in a virtual environment. There has been other research that focuses on wearable necklaces (Amores and Maes, 2017) or clips (Amores et al., 2018) for wellbeing. Other neck-based wearables have been used to augment mobile notifications by Dobbelstein et al. (2017), and more recently, Lin et al. (2020) created a breath training toolkit that uses a wearable scent-emitting device that is activated with a stress ball. Last but not least, in this article we present an olfactory wearable that can be worn during the day and night coupled with a brain activity sensor to predict sleep stages and release scent at certain stages inprecise quantities.

#### 2.3.4. Intra-Nasal Methods in Medicine

Nasal cannulas and nasal masks are the most common method for scent delivery in hospitals, clinics, or specialized care facilities. Although they are “wearable,” they are connected to stationary olfactometers that are computer-controlled and can determine the airflow, odor concentration, and odor duration. Therefore, this method is probably the most efficient to use for stationary, clinical settings but they are challenging to deploy while walking or at home. The nasal cannula delivers small quantities of scent directly to the nasal cavity through the nostrils, and scent lingering in the surrounding space is minimal. This is especially relevant for intranasal medication delivery since it is the fastest way to absorb molecules after intravenous methods (see Patton and Byron, 2007). Close-to-nose interfaces could be used as a complementary or alternative wearable method to nasal cannulas, alleviating the discomfort caused by these intra-nasal devices.

### 2.4. Biological OI

A “Biological” scent-delivery method is emitted through or within the person. For example, artist (McRae, 2011) created a speculative design fiction called “Swallowable Parfum,” a cosmetic pill that makes the skin perspire a fragrance, therefore using the body as an atomizer. Kan et al. (2017) created pH-reactive materials that can change color, odor, and shape. Amongst many other applications, these materials can be applied to the skin and change scent based on sweat or tears, for example, by sensing lactic acid present in sweat. Other biological scent-delivery methods include “bacterial” scent-delivery (delivery of odors using synthetic biology). Finally, biological scent-delivery can include scents that are digitally generated using electrical stimulation or optogenetics. These types of scents are perceived as “real,” although they do not exist physically. This exciting research is still under-explored and remains mostly unknown, although some intriguing work has been done in this area, predominantly in rats by Mouly and Holley (1986) and Chong et al. (2020). Other exploratory research in humans has been done by Kumar et al. (2012) and Holbrook et al. (2019). Even though these studies are promising, the methods used are still invasive and require significant development before we can arbitrarily create synthetic scents on demand.

### 2.5. Passive/Active OIs

Besides the interaction of odor in space, scent can interact with the user in an active or passive manner. We focus on the use of scent in the sleep environment and refer to “Sleep interfaces” or “Sleep UI” as technological interventions in which a person controls a software application or hardware device that can trigger a stimulus like scent while sleeping. These closed-loop systems can monitor physiological signals from the user and generate an output such as a burst of scent, audio, tactile stimulation/feedback, or a light-based stimulus.

#### 2.5.1. Active Sleep UI

We consider an “active” sleep UI a closed-loop user interface that automatically changes according to the user's sleep stage, similar to the system presented in this article. There are other ways to interact with the user during sleep, e.g., using visual, tactile or transcranial magnetic stimulation (which have been used to trigger lucid dreams, see the work by LaBerge and Levitan, 1995; Paul et al., 2014; Voss et al., 2014). Commercial devices such as Night Shift would also fall into this category, by preventing snoring and obtrusive sleep apnea by making the user change their position using haptic feedback. Dormio (Haar Horowitz et al., 2018) tracks when the user is falling asleep (hypnagogic state) and plays a sound or keywords to influence micro dreams in the first stages of sleep.

#### 2.5.2. Passive Sleep UI

Sensorwake is a commercial product that releases bursts of scent to wake people up (some of the scents used are mint, which will activate the trigeminal nerve, in contrast with the odors that we use in this article). The device does not detect any sleep stages or any input from the user; therefore, the user does not “control” it. Thus, it might not strictly be named an interactive or closed-loop “sleep interface,” but can instead be categorized as a “passive” sleep UI. Therefore, one might also categorize a sound alarm clock as a “passive” sleep UI since the user does not control it, but the interface will influence the sleeping mind. Similarly, any type of input while sleeping could be categorized as a “passive” sleep UI, such as a humidifier, the bed's stiffness, the texture of the bedsheets, etc. Although the definition of a “passive” sleep UI might seem too broad at first, it could open up new opportunities and ideas to think about how they could transition into an active/interactive or closed-loop sleep interface.

## 3. Design and Implementation of Ezzence

The goal of our research is to develop an OI that is portable, can be used during wake and sleep state, does not wake the user up, is easy to use and setup at home and can be worn in an ambulatory context during the day. Therefore, our aim is to develop and evaluate an olfactory device that in comparison with previous **HCI** scent-delivery systems:

- Is designed for interactions that extend from wakefulness to sleep.
- Can be deployed in remote human subject studies in which participants sleep in their homes.
- Can release scent in real-time based on specific sleep stages, which is key for applications such as targeted memory reactivation.
- Is modular so it can be worn in many different ways and attached to different surfaces for maximum flexibility and comfort.
- Can release a burst of scent with a duration faster than 1 s, which is important for certain applications.
- Is designed for longitudinal studies.

In comparison with olfactometers used in **scientific sleep studies**, the prototype presented here is:

- Over 700 times smaller and 40 times lighter than the smallest PSG olfactometer (Risso et al., 2018).
- Mobile and usable during sleep and wakefulness.
- Silent enough to be used in the same room as the sleeper without disturbing their sleep.
- Does not require a nasal cannula/mask.
- Controllable wirelessly so that scent release can be customized via a smartphone app.
- Tested outside of a research laboratory in a natural, home context.

As far as we are aware, our device is the only silent wearable olfactometer that precisely releases scent down to microseconds (μs), is designed to be used for both sleep and wake interactions, can release multiple scents, and integrates an automatic sleep staging scent release mechanism. Our device uses ultrasound atomization which has been used in the past and is one of the most silent ways of releasing scent, which is critical for sleep applications. We use piezoelectrics that vibrate at a high frequency to realize a minimum delay between activation and arrival of the scent to the user in comparison with systems that use heat or fans. Our goal was to design a device that people could use comfortably at home, that was silent, portable and that could also be accurate enough for scientists to use in sleep experiments. Given the burden, cost, and difficulties of deploying olfactometers for sleep applications “in-the-wild,” sleep research in home-based settings are scarce (we were not able to find any at-home sleep study using these type of technologies).

### 3.1. Technical Description

**Figure 3:**
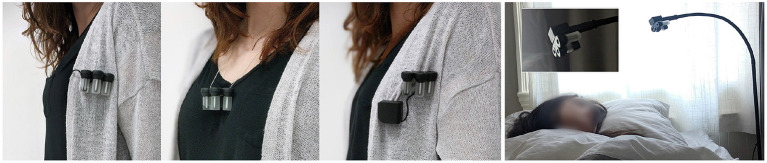
*Ezzence* can be worn during the day and be attached to a bedside holder at night. It can also hook onto pajamas, blankets, necklaces or, inspired by a brooch; each part can be attached separately. The custom holder charges the device and has an adjustable flexible neck. It wirelessly connects to the smartphone application that controls the duration and frequency of the scent bursts and activates odors depending on physiological signals, sleep stage, and the user's preferences.

**Figure 4:**
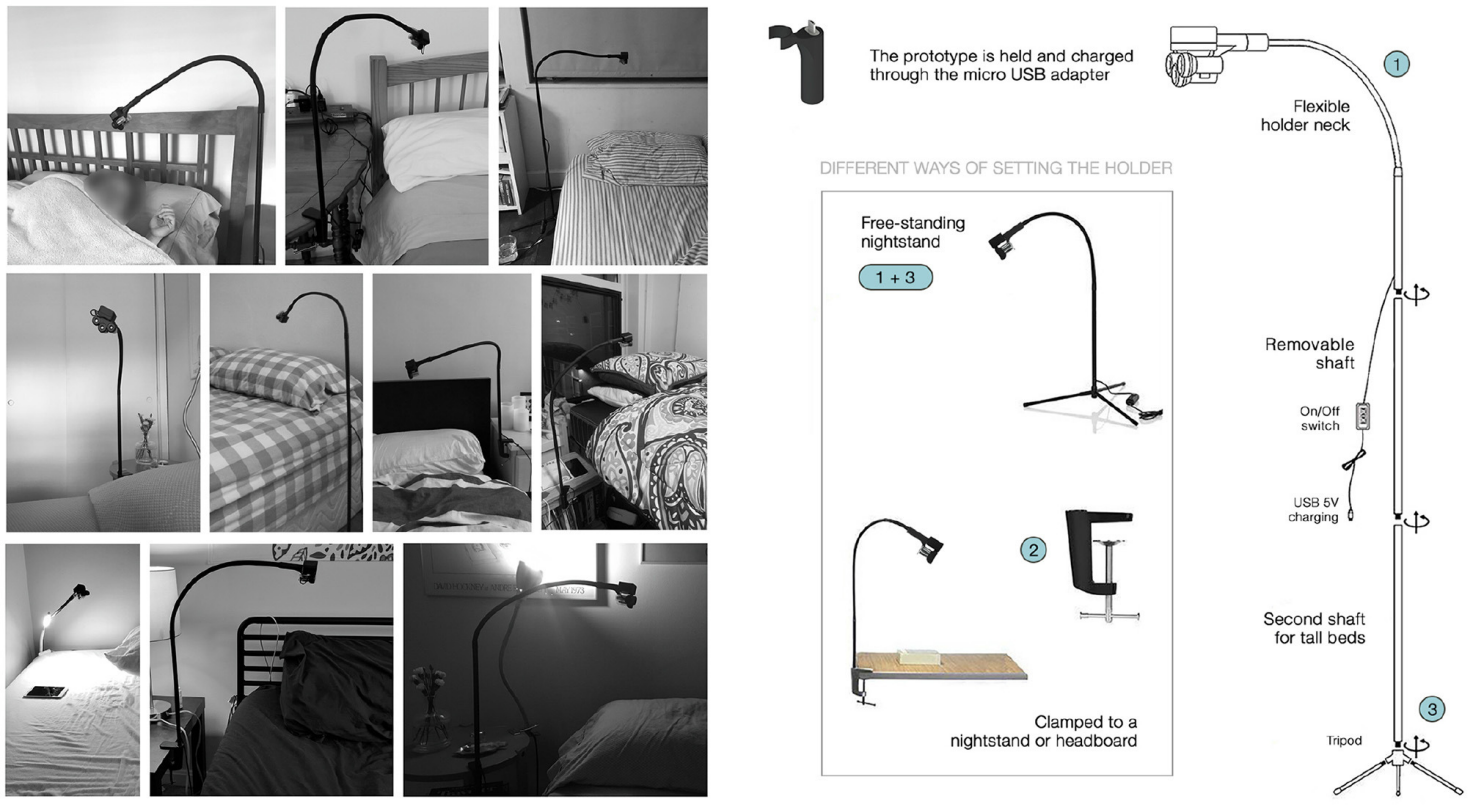
The “sleep holder” charges the device while the user sleeps. The distance between the prototype and the user's nose can be adapted via the flexible holder head (1). The user can assemble the holder clamped to the nightstand (2) or like a tripod next to the bed (3). On the left, pictures are taken by participants after assembling the prototype to the holder in their homes.

**Figure 5:**
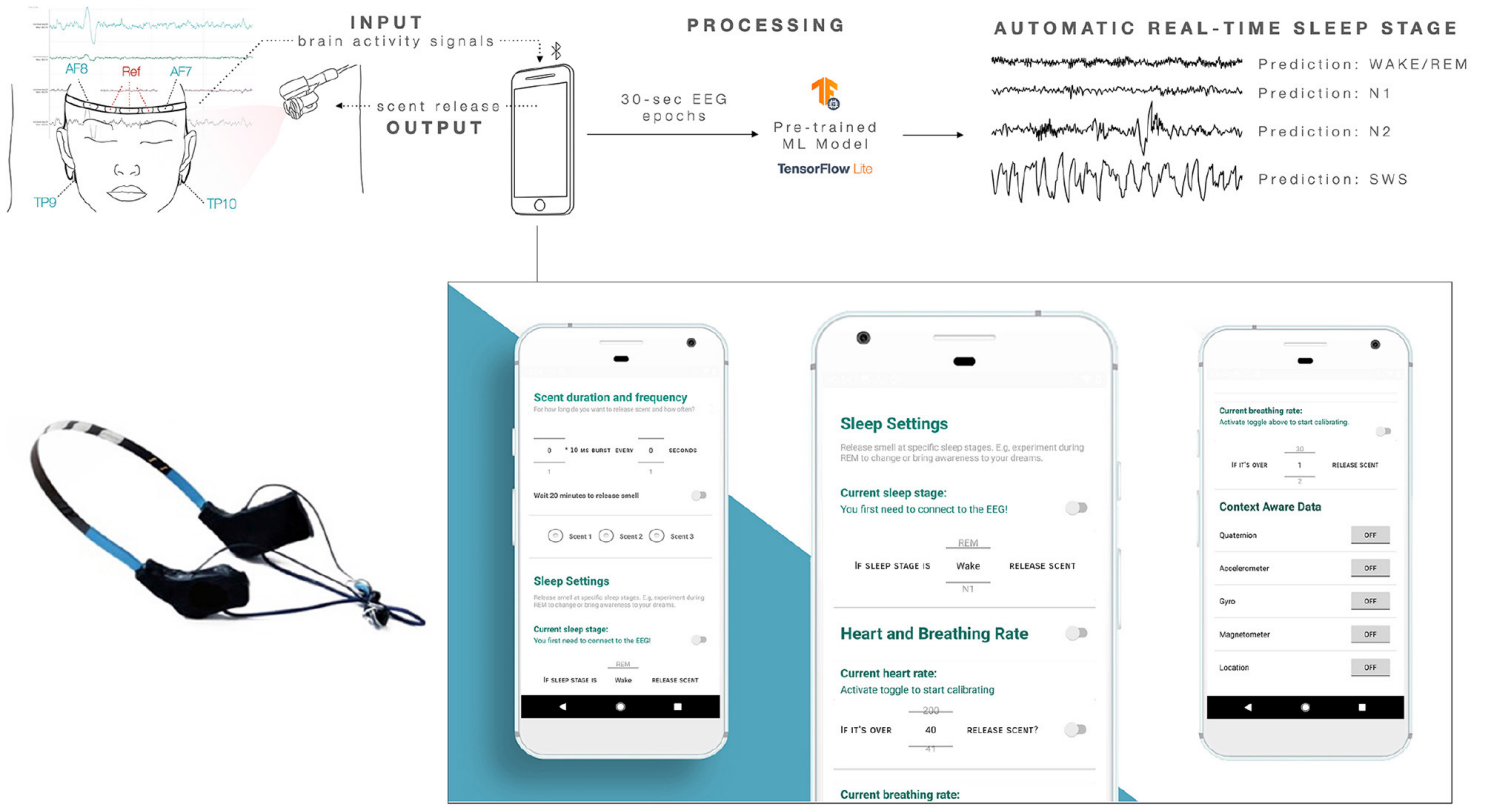
The system reads brain activity signals and sends this information from the EEG to the smartphone via Bluetooth Low Energy (BLE). The app integrates a pre-trained machine learning model that takes 30 s worth of data from the EEG and predicts a sleep stage with a given accuracy. The user or experimenter can customize the duration and frequency of the olfactory stimulation in certain sleep stages using the smartphone app. Scent can be activated manually using a button, or automatically based on physiological signals, such as heart rate and breathing rate. The system can also release scent based on the user's sleep stage, or based on time, e.g., 20 min after going to sleep.

**Figure 6:**
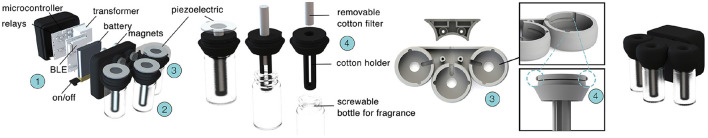
(1) The back case encapsulates the PCB and connects to the front case (2) via a textile thread and magnets. The case holds the piezoelectrics and bottles and has a cavity that connects the textile thread to them (3). The piezoelectrics vibrate at high frequency and release scent when in contact with the cotton filters by soaking the fragrance from the bottle (4). The injection-molded case holds the magnets and wires with two screws. The fragrance can be refilled in two ways: one is by unscrewing the bottle of glass, and the other is by replacing the entire capsule (including cotton filter and cotton holder). The capsule is held using two nubs (4) with an internal mechanism that locks in place by inserting it upwards while rotating 60° (until the nub passes a minor bump in the path).

We designed a prototype that can automatically release scent ranging from microseconds to seconds, generating minimal delay between the activation and arrival of scent to the user. It can be configured prior to sleep via a custom made Android App (see **Figure 5**), in which the user (or an experimenter) can customize the burst duration and timing of multiple scents. The device can release up to three different odors simultaneously, as shown in Figures 3, **6**. The user/experimenter can preset triggers and rules for real-time scent release based on changes in sleep brain wave activity (as shown in **Figure 5**), as well as physiological data. The app allows the user to select at what sleep stage scent should be released. If worn on the body, the device can also detect heart rate and respiration and be programmed to release scent accordingly (Amores et al., 2018). Scent can also be delivered based on the user's location and accelerometer information from the phone. The prototype can be adapted to the user and experimenter needs and bedroom settings (see Figure 4). The design is modular so that users can wear it according to their preferences. The aim was to design a unisex device that could be portable or wearable, and most importantly, the device had to be close enough to the nose so that small doses of scent could be released into the air without habituating the user. A friendly user interface also had to be created to configure the device easily so participants could use it at home.

### 3.2. Smartphone App and Real-Time Sleep Staging

An Android smartphone app that connects to the olfactory device via Bluetooth Low Energy (BLE) using UART communication was developed. In the app, the user can control how often and how much scent is released. In “Sleep Settings” (see Figure 5), the user can connect to the Muse EEG, so that brain activity information is streamed. The hardware of the EEG is a modification of the original Muse headband by Interaxon. This headband was designed to be used for meditation, not for sleep. Interaxon has recently launched a new EEG headband “Muse S” for bedtime usage, but there is no available API or service to stream sleep stages in real-time. Therefore, the previous SDK and the original Muse headband to stream real-time brain activity were used. The original casing of the EEG was modified to replace Tp9 and Tp10 electrodes with electrodes attached to the skin for better comfort and robustness of the signal while sleeping.

The smartphone app integrates a machine learning model proposed by Supratak et al. (2017) and Koushik et al. (2019) to infer sleep stages in single 30 s epochs by using Time-Distributed Convolutional Neural Networks (CNN) and just one electrode using the Muse EEG headband. The system's accuracy in predicting four sleep stages and wake was reported to be 83.5% on 20-fold cross-validation using the open Sleep-EDF dataset by Kemp et al. (2000). For comparison, inter-rater reliability among sleep-scoring experts is about 80%. A new model was generated for this article with data from 20 participants (the training-set is 40 nights in total). The Tensorflow Lite model was imported to the Android App, and a UI was created to let the user pick at what sleep stage scent should be released. When the toggle is activated, the previously set intensity and frequency will be active, and the scent will be released when the accuracy of the predicted sleep stage is over 60%.

### 3.3. Hardware

The device consists of two main structures (1) and (2) (as depicted in Figure 6). They are connected via a textile cable thread and can be magnetically attached and worn with a layer of fabric in between (shown in Figure 3). Rare-earth magnets were chosen for their strength and to allow for versatile use of the device.

#### 3.3.1. Electronics and Back Case

The dimensions of the electronics back case are 3.3 cm (L) × 3.8 cm (H) × 2 cm (W) (see Figure 6). It has two mounting magnets designed to attract the front case and an on/off piece attached to the PCB. The PCB design was based on previous work (Amores et al., 2018), with variations in the type of piezoelectrics used, transformers, and battery to minimize power consumption and maximize the efficiency and speed of the bursts.

Previous work uses a piezoelectric with a 16 mm diameter, 110 kHz frequency, and an unknown size of microholes. Instead, we were interested in testing different sizes of microholes, which would allow the use of more viscous essential oils. Additionally, controlling the size of the microholes is crucial as it will vary the amount of fluid and power per burst of scent released. For this reason, we visited a piezoelectric company and were able to create our own custom microporous sizes. We created various microhole sizes (e.g., 10, 12, 13 μ) based on the recommendations and limitations given by the manufacturer. Finally, we chose 13 W, 4.5 D microholes with a frequency of 108 kHz (compared to the 110 kHz). These piezo parameters were our preferred after volatilizing higher liquid viscosities and emitting longer bursts in shorter times. Additionally, we updated the transformer, we currently use a 10:0.45 transformer (0.045, Ratio 20:1) instead of a 5.4:0.5 transformer (0.09, Ratio 10:1); thus, the prototype is more efficient than in previous work, taking 24 mAh when the BLE is connected and 250 mAh when a burst is released (instead of 60 and 500 mAh). Such intensity is due to the high voltage needed to run the piezoelectrics at a high frequency of 108 kHz. Finally, compared to previously used batteries (350 mAh), we use a smaller battery, almost half the weight and height (150 mAh).

#### 3.3.2. Capsule Holder

The capsule holder consists of a glass bottle (which can be easily unscrewed and refilled), a cotton holder, and a cotton filter that transports small quantities of liquid through its fibers to the piezoelectric (as shown in Figure 6). The cotton filter is supported from the bottom to apply enough pressure for the piezoelectric to release scent (3). Minimal changes in the pressure of the piezoelectric can prevent the scent from being released. Different designs were carefully studied and tested until the best performance was achieved (e.g., the bottle holder has cutouts for the liquid to be absorbed from the side and bottom). All components were custom made, we tested different stick filter hardness, lengths and diameters (some of the results shown in Table 1). It was found that diameters of <5 or 29 mm long had leaks (e.g., 4 and 28 mm). Lengths larger than 29 mm excessively pressured the piezoelectric and would not release scent and sometimes break the disc. Thus, the final settings used for the study were those with the longest-lasting time and no leaks that were achieved using a rigid stick filter with a diameter of 5 and 29 mm long.

**Table 1:** Test conditions for 10 ms bursts every 60 s.

**Substance**	**Cotton**	**ml/burst**	**Lasting time**
H2O	Rigid	≈ 0.0023	≈ 15 h
H2O	Soft	≈ 0.0031	≈ 11 h
**80% H2O + 20% Sleep blend oil**	**Rigid**	≈ 0.0006	≈ 54 h
80% H2O + 20% Sleep blend oil	Soft	≈ 0.0014	≈ 27 h
**90% H2O + 10% odorless oil**	**Rigid**	≈ 0.0007	≈ 46 h

*The table depicts the average burst sizes for various substances and cotton filter densities and their calculated lasting hours (using a 2 ml bottle)*.

#### 3.3.3. Front Case

See Figure 6 (2): The front case has two magnets with reversed polarities to the electronics back case (1). A rotate and snap mechanism was designed to make the refill and replacement of fragrance simple, quick, and clean. Users can choose between replacing the capsule with a new one (including a new cotton filter), or refilling the bottle.

#### 3.3.4. Piezo Holder

See Figure 6 (3): The piezoelectric disks are placed on top of the cotton filter. Separate piezoelectrics cavities were designed to reduce cross-contamination between the capsules.

#### 3.3.5. Sleep Holder

The prototype holder was designed to clip to a bedboard or nightstand (as showcased in Figure 4). The inspiration came in the form of a gooseneck night light that was modified to hold and charge the prototype. The gooseneck provides flexibility to adjust the distance between the prototype and the user's nose, regardless of their bedroom layout. The holder is 3D printed and holds the prototype in place (even upside down) with a plugged-in micro USB and a snap clip that holds the center bottle on the front case. The snap clip was tested and iterated upon so the prototype can be easily inserted and removed without breaking.

#### 3.3.6. Case Materials and Design for Manufacturing

The casing was initially 3D printed using a Form 2 with multiple types of Photopolymer resin as well as with Stratasys. Unfortunately, essential oils and alcohol-based fragrances are corrosive and eroded the cotton holder and caused the case to change color, bow, deteriorate, and fail after a couple of months of using the prototype. With the need for a durable material and to accommodate the expected number of devices for future user studies, an injection mold case was fabricated. We manufactured ~100 PCB boards and 100 injection molded cases. The materials used were HDPE and PP (commonly used in the food industry) with different colors (white, black, and transparent). The black color was chosen for the final design based on the users' feedback to avoid the LED light shining through the case at night.

### 3.4. Technical Tests

The prototype was tested for multiple nights in a row, with various substances, ranging from essential oils, hydrosols, to water with different carrier oils. The results shown in Table 1 are the chemical compounds used for the pilot and final study (in bold). The variables involved in how long the scent can last in the prototype are: *f(n, c, X, Tb, Fb, B)*, where *n* = number of containers, *c* = container capacity in ml, *Tb* = burst duration in ms, *Fb* = how often scent is released (e.g., every 60 s) and *B* = burst size in ml/ms. *B* is a system-dependent parameter that depends on the size of the piezoelectric microholes (larger holes will create bigger burst, therefore larger quantities of the liquid will be emitted), the viscosity of the liquid in cps (centipoise units), type of cotton filter (softer cotton creating a bigger burst), and the pressure between cotton and the piezoelectric. The battery power will also influence these results, as well as natural variability in injection molding, which affects the way the holder and cotton presses against the piezoelectric.

#### 3.4.1. Cotton Filter, Piezoelectric, and Viscosity

It was found that the olfactory user experience varies depending on the cotton filter and the piezoelectric. High density filters have slower absorption and volatility than low density filters [soft cottons release more scent, harder (more rigid) cottons emit less quantity]. The same holds for a piezoelectric with larger microholes (more odor), while a piezoelectric with smaller microholes will release less. It was also observed that with a high viscosity liquid (e.g., CBD oil), the atomization performance worsens after a few bursts; therefore, it is recommended for future researchers to dilute the fragrance based on the centipoise units (cps) of the liquid's viscosity.

#### 3.4.2. Dilution

Previous researchers (Diego et al., 1998), conducted their studies using essential oil diluted at 10%. Based on their insights, similar ratios of fragrance were tested. Water was chosen as a diluting substance because a carrier oil like coconut oil was too viscous to be atomized and resulted in smaller bursts that did not reach the participant's nose. The ideal ratio for the essential oil selected (“Good Sleep Blend” by Solutions, 2021), was 20% essential oil and 80% water. A variety of essential oils diluted in water at different ratios were tested at a distance of ~ 20 cm away from the participant's nose using the “sleep holder.” Several odorless carrier blends of different viscosities were tested to use a similar viscosity for the control group than for the experimental group (using the sleep blend). The blends consisted of different ratios of Benzyl Benzoate (≈9 cps) and Isopar H. (≈1.5 cps) to achieve blends with viscosities of 2, 3, 4 cps, etc. The prototype was tested with several of these carrier oils at ratios of 20% oil and 80% water, but unfortunately, they clogged relatively fast (after <30 min of usage). With further testing, the ideal ratio of 10 and 90% water with a 2 cps viscosity was found.

## 4. Materials and Methods

### 4.1. Evaluating the Use of Ezzence

To evaluate the usability and feasibility of the Ezzence device for home-based sleep studies, we conducted a user study with 40 participants. We opted out for only assessing the usability and feasibility of Ezzence without the EEG sensor to avoid confounders that could have biased the study (e.g., decreased sleep quality due to the head-mounted sensor instead of the Ezzence device).

The study was conducted using a between-subjects experimental design. To avoid observer-expectancy effect and unconscious influences on participant behavior, half of the participants were randomly assigned to the control condition (prototype with release of water) and the rest to the scent condition (lavender blend). Non-smoking, healthy participants were recruited by an e-mail that was sent to the department (21 female, 19 male, with a mean age of 28.7; the oldest person was 56 years old and the youngest 19). No one suffered from respiratory problems or odor allergies. A written, informed consent form was obtained from all participants before participation, and the university ethics committee approved the experiment.

### 4.2. Daytime Olfactory Test

Participants were given the consent form, filled out a survey about their demographics, the dimensions, and ventilation of their bedroom as well as their habits related to the use of scent while sleeping. After that, they performed an olfactory test and were told how to set up the prototype. The total duration of the olfactory test was ~30 min, and its primary goal was to:

1. Determine if users perceived a 10 ms burst of scent.
2. Evaluate how strong and pleasant they perceived it to be.
3. Identify if there were participants that had anosmia or difficulties smelling, so they could be removed from the study.
4. Test the usability and efficiency of the rotation mechanism designed to replace and refill the scent containers.

**Figure 7:**
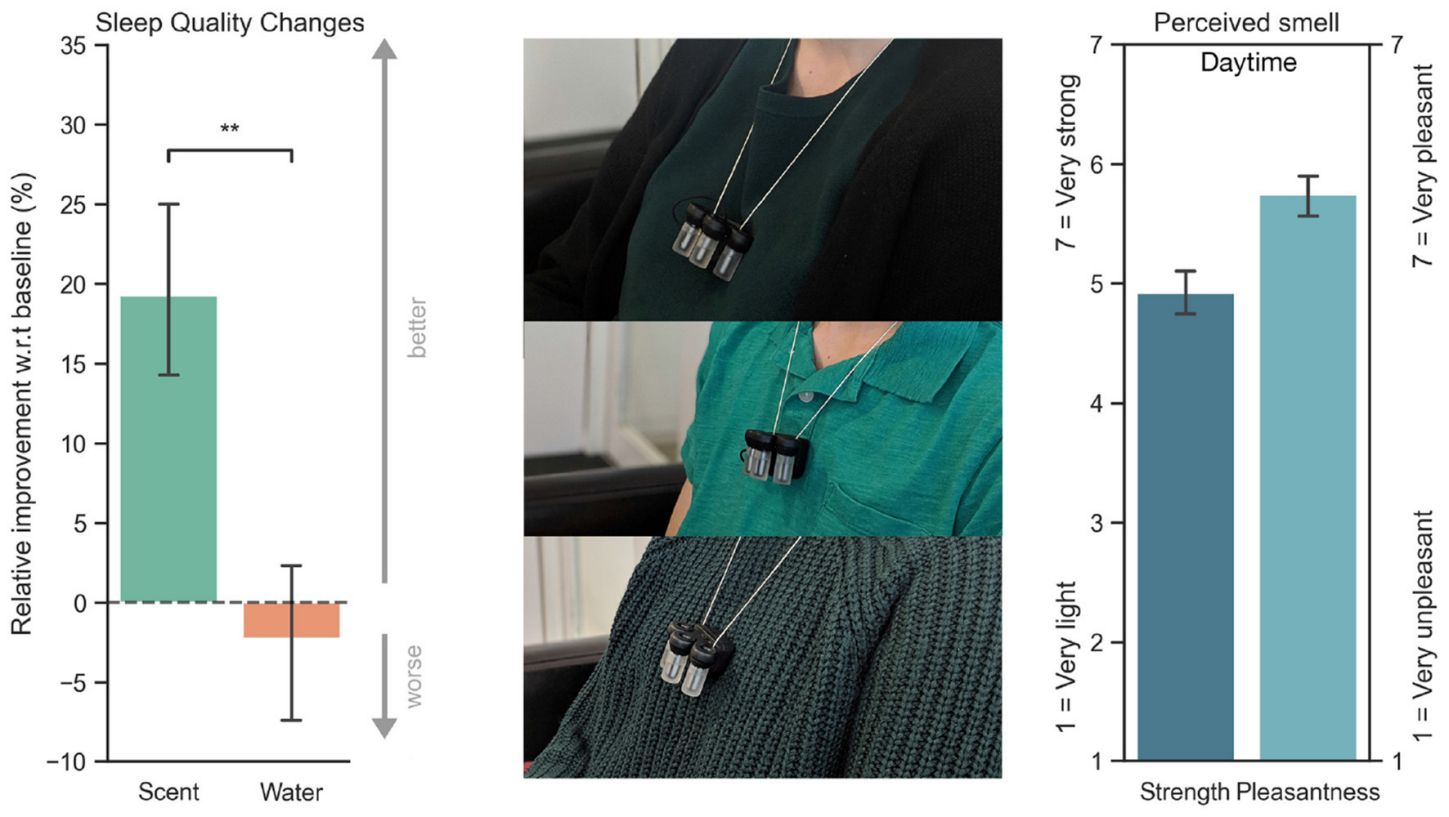
Participants wearing the prototype during the daytime olfactory test. As seen in the right image, participants rated how strong (1 = Very light, 7 = Very strong) the fragrance was, as well as how pleasant (1 = Very unpleasant, 7 = Very pleasant). On the left, self-reported relative improvement with respect to their sleep quality on a baseline night. There was a significant improvement when comparing scent with control (water). **P < 0.01.

During this daytime test, participants wore the prototype as a necklace shown in Figure 7 and inhaled diluted essential lavender mix oil at a signal given by the experimenter (only then a 10 ms burst was released). They were asked to report if they could smell something, for how long, how strong, and how pleasant it was. After this, five control (80% water, 20% odorless carrier oil) and five diluted essential lavender mix oil were randomly triggered every 30 s (with pauses in between). The rationale for the 30 s was based on previous work on habituation and desensitization (Sinding et al., 2017). Finally, we tested the usability of the refilling mechanism. Participants were not required to refill the bottles in our sleep study because it was only one night. However, we wanted to test the usability and ease of refill for potential future use cases where participants might need to take the device home for several weeks. Thus, we conducted a practice test in which we measured the time spent by participants filling the scent container and their subjective feedback.

### 4.3. Preparation for the Sleep Study

Participants were told that the study's goal was to deploy the device in their homes to test its usability. They were not given any instructions about how it might positively/negatively affect their sleep and mood the following morning. They did not know if they were assigned to the scent or the control condition. They were instructed to sleep at least 7 h and not consume alcohol or caffeine after 3 p.m. (as suggested in the literature). They were asked to keep their pets outside of their bedroom and make sure that, if they shared the bed with someone, that person did not have odor allergies or respiratory problems. They were given instructions to use the device and the smartphone app. They learned how to set up an automatic scent release of 10 ms every 60 s that would be triggered 20 min after falling asleep. They were given instructions to assemble the holder and adjust the flexible neck (so that the prototype faced toward their nose, approximately two hands away from their face ~ 20–40 cm). They took the device, the holder, and a document (with all the detailed steps) to their homes and slept with the device for one night. In the morning, they filled out two surveys: one about their sleep quality and the functioning of the prototype (right after waking up) and another about their overall experience with and usability of the device. Some days after the study was finished (without prior notice), participants were asked to report their sleep quality for a typical night.

### 4.4. Odorants and Delivery

The scent selected was a commercially available essential oil blend “Good Sleep Blend” by Solutions (2021) that contains Lavender, Clary Sage, and Copaiba oil, and has a viscosity of 3.9 cps. 20% of the sleep blend oil was diluted in 80% of distilled water. The fragrance was chosen based on its high ratings on pleasantness and lavender's natural sedative effect (Diego et al., 1998; Motomura et al., 2001; Goel et al., 2005; Lehrner et al., 2005; Field et al., 2008; Yazdkhasti and Pirak, 2016). Distilled water with 10% odorless carrier oil was used for the control group. All odors were delivered at low, non-trigeminal concentrations by the device that was worn on the chest during the olfactory test and in the holder near their nose in their bedroom at night (as shown in Figure 4). The devices were cleaned and replenished after each use. All sleep experiments were conducted in people's homes, without the experimenter's intervention or presence. Scent or water was released 20 min after they reported lying down to sleep (20 min after they triggered the sleep timer on the smartphone UI to “on,” see Figure 5). Scent or water was triggered at a frequency of 10 ms of burst every 60 s. The main reason behind this frequency was to reduce the length of the burst and minimize the number of molecules released in the air to avoid habituation (also known as olfactory fatigue, or “nose blindness,” see the work by Chaudhury et al., 2010 for more information on this topic). This frequency was also used to ensure that it might overlap with some K-complexes and sleep spindles (McCormick et al., 1997; with the hopes to increase deep sleep/slow-wave activity). The bottles contained 2 ml of liquid (enough to last for an overnight of sleep without refilling).

### 4.5. Data Exclusion and Technical Problems

Data from 10 participants was excluded from the analysis. Of these, three participants did not finish the study. Two of them because of work deadlines/other personal issues, the other because the participant had a diminished sense of smell because of a cold. The seven other removed participants (one male, six female) reported that the prototype worked but that they did not see a “burst/scent” in the morning due to the following reasons: one participant set up 10 ms of scent every 6 s instead of every 60 s, therefore running out of fragrance earlier than expected. Another participant reported plugging the prototype into an outlet that did not work and realized it in the morning; therefore, the prototype stopped working 5–6 h after the first burst (using the internal battery instead of external power). Similarly, another participant reported not turning the switch on. The fourth participant reported “bumping into it” and suspected that she probably unplugged it. Another participant set up the wrong frequency as when the prototype was returned, there was no liquid left. Finally, the last participant reported that the prototype might have fallen, or he might not have charged it properly.

### 4.6. Statistical Analysis

To test if the differences between the scent and the control group's means were statistically significant, we first examined if the assumptions to perform a two-sample *t*-test were met: normal distribution of data, homogeneity of variance, and independence of the observations. Each participant belonged to only one group (either scent or control group measured with interval scale values). Thus, there was no relationship between the observations in each group. For sleep quality changes (Figure 7), both the control and scent conditions had fairly symmetrical data [slightly positively skewed for the scent condition (0.4) compared to control (−0.1)], both distributions had a short-tailed distribution (Kurtosis of −1.7 and −0.8). To test if the variance of the outcome variable was equal in each group, we applied the Levene test (*p* = 0.199). The variance criterion held true (*p* > a), where “a” is the probability threshold set to 0.05. As *p* > 0.05, the data sets conform to the variance criterion. In addition, we computed the Shapiro–Wilk test for each group [*p* = 0.229 (control condition), *p* = 0.01] for the scent condition. The results showed that only the Control group had *p*-values greater than the significance level 0.05, indicating that the data distribution is not significantly different from the normal distribution. In summary, based on the results from the Shapiro–Wilk test, we can only assume normality for the control group. Thus, we ran both parametric and non-parametric tests to validate our results because the scent group was only moderately skewed. In addition, the parametric *t*-test has been shown to be robust against non-normality, and some argue that there is nearly no need to use Wilcoxon (Mann–Whitney) non-parametric test (Rasch D. et al., 2007). Nevertheless, for completeness, we ran both tests. We demonstrated that both tests show a statistically significant difference between the means of the two groups (scent vs. control): Mann–Whitney-Wilcoxon (*p* = 0.009) and independent *t*-test (*p* = 0.003), both one-tail. Similarly, we also ran both tests for the perceived mood, rest, deep sleep, awareness, etc., and found that they resulted in very similar *p*-values, with both significant results for sleep onset (*p* = 0.010 for Mann–Whitney and *p* = 0.008 for the *t*-test), mood (*p* = 0.043 and *p* = 0.038, respectively), and scent awareness (*p* = 0.001 and *p* = 0.005). Neither of the tests showed a significant difference between the scent and control conditions for the perceived rest, deep sleep, and positive dreams. The Supplementary Material provides more details on the analysis, the Python code, and data, including the original quotes from participants with their feedback.

## 5. Results

All the prototypes successfully worked as expected, except for seven participants that reported not turning on the prototype, unplugging the device, or setting the wrong frequency. Nobody dropped out of the study. All participants from whom the data was analyzed reported seeing a burst of scent/water coming out in the morning. They did not have any problems or sounds due to malfunctioning, although two participants returned the holder broken, both because of transportation/packing or falls. One participant tried to unscrew an internal part of the holder; the other cracked the 3d printed neck (both when trying to pack it and return it to the laboratory).

### 5.1. Daytime Olfactory Results

Participants' responses regarding how long they smelled the 10ms burst during the olfactory test were the following: 33.3% noticed the smell for only 1 s, 13.3% for 3 s, 26.6% for 3 s or more, and 26.6% for 2 s. In terms of how strong (1 = Very light, 7 = Very strong) the M = 5.03; SEM = 0.21. The results for how pleasant (1 = Very unpleasant, 7 = Very pleasant) were M = 5.8; SEM = 0.19. Participants successfully differentiated between placebo or scent with an average of 8.81 correct responses out of 10, SEM = 0.16. All participants could refill the bottles; the average time spent refilling one of the capsules was ≈28 s, SEM = 1.03.

### 5.2. Night Time Awareness and Awakenings

**Figure 8:**
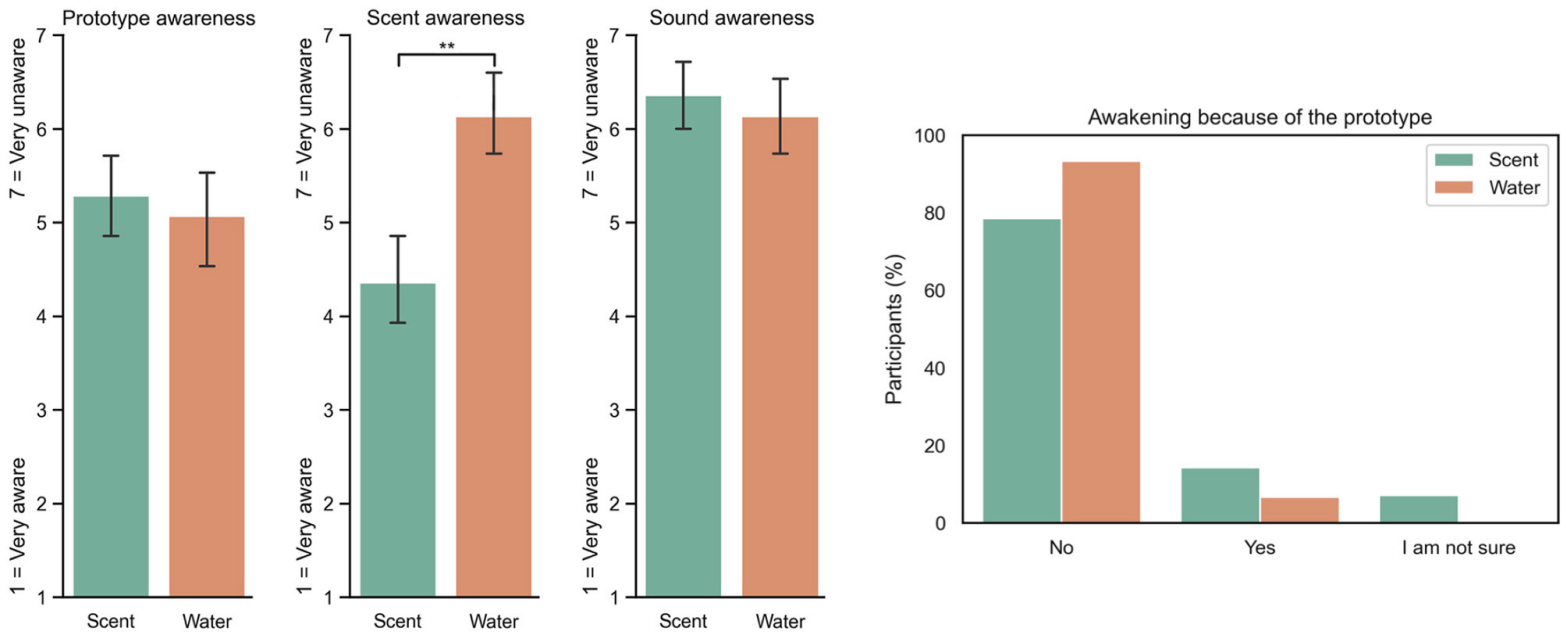
Answers to the questions “How aware were you of the prototype?”; 7 = Very unaware, 1 = Very aware, a neutral response is a 4. “How aware were you of the scent?" and “Did you wake up because of the prototype?” Ninety-three percent of participants that were in the control condition and 78% in the scent condition reported not waking up because of the prototype. **P < 0.01.

Overall, participants were unaware of the device and remained mostly neutral to unaware of the scent while sleeping, as seen in Figure 8. There was no significant difference between the scent and water groups in the case of prototype awareness and sound awareness (disturbing noises from the apparatus). However, there was a significant difference in the scent awareness in the case of control vs. scent. The results suggest that participants remained neutral to slightly unaware of the scent and unaware in the case of water. Ninety-three percent of participants in the control condition and 78% in the scent condition did not report awakenings due to the device. Therefore, even though most participants did not wake up, there was some level of olfactory awareness that they were able to report. It is unclear why this might be the case, but one hypothesis is that they could smell the fragrance in a state of drowsiness or hypnagogia in the early transition into sleep/stage 1, as shown by previous work by Carskadon and Herz (2004). Most sleep studies track physiological information, such as EEG, to recognize arousals. However, they usually do not ask questions about the level of awareness or subjective olfactory experience after sleeping. This suggests that although most people do not wake up and remain asleep, some participants seem to be semi-conscious of the scent.

### 5.3. Perceived Sleep and Mood

**Figure 9:**
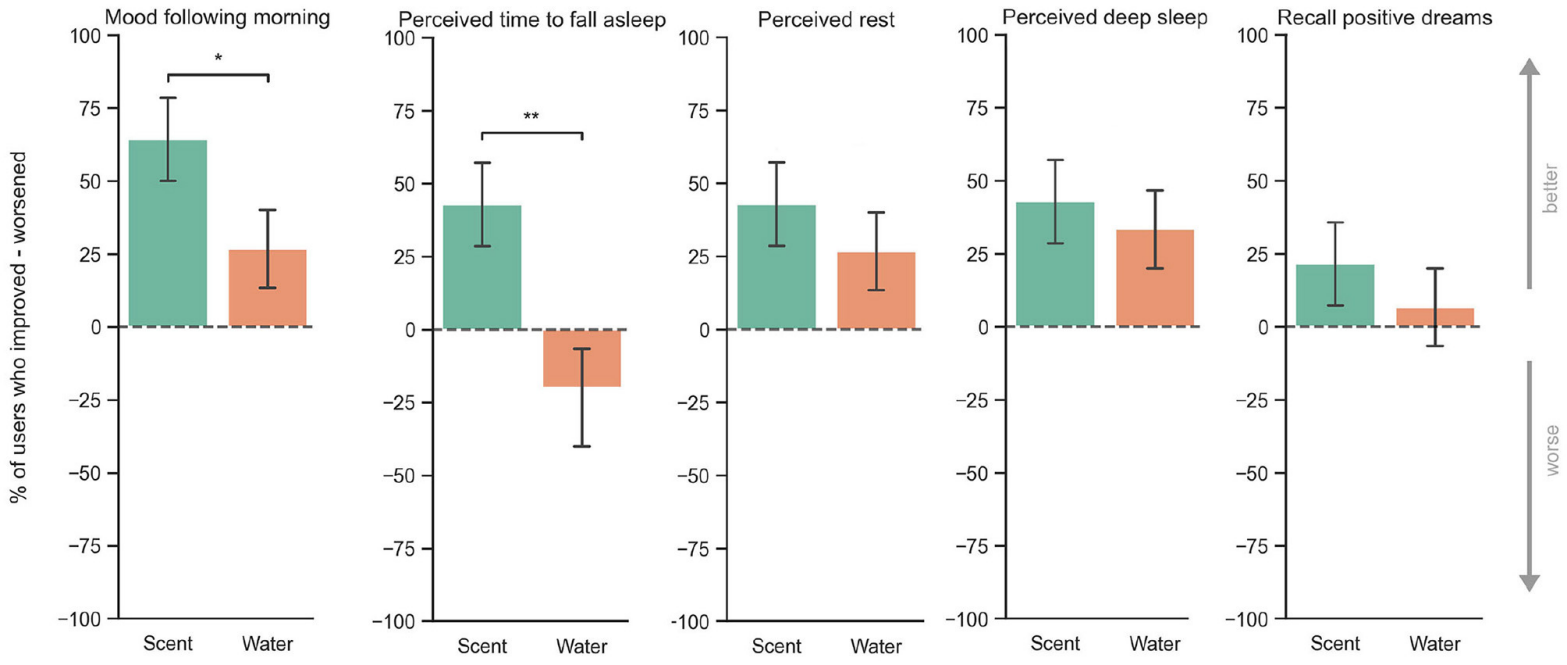
In turquoise, experimental condition using the prototype with a lavender-based essential oil. In orange, prototype with water. The bars depict the percentage of participants who reported improvement minus those participants who reported worsening their sleep. Participants that were in the scent condition had on average more improvement than those in the control condition (water). *P < 0.05, **P < 0.01.

On average, all participants that used the prototype with scent vs. those in the control condition, increased their perceived sleep quality, depth of sleep, perceived rest at night and increased the content of positive dreams as well as their mood the following morning, and decreased their time to fall asleep. Depicted in Figure 7, the results of an independent t-test show that participants reported a significant improvement in their sleep quality changes when compared to their typical sleep when using the device with scent in comparison to the control condition [*t*_(27)_ = 2.9, *p* = 0.003 (one-tail)]. As shown in Figure 9, there was no significant difference in the case of perceived rest (*p* = 0.22), deep sleep (*p* = 0.30), and the recall of positive dreams (*p* = 0.25) but there was a statistically significant number of users that reported better mood the following morning in comparison to control (*p* = 0.038), and they were also significantly faster to fall asleep (*p* = 0.008). Sixty-eight percent of Confidence Intervals (±1 STD) and one-tail *p*-values are reported.

### 5.4. Usability and Qualitative Feedback

**Figure 10:**
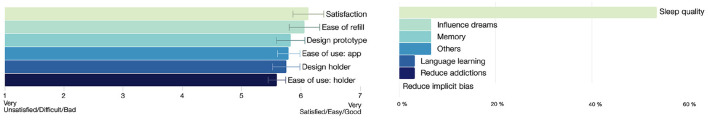
**(Left)** Usability results for the prototype where 7 is “very positive” and a neutral response is a 4. Satisfaction using the prototype. **(Right)** Answers from all the participants to the question “What would you prefer to use the prototype for?”

Participants rated very positively the overall satisfaction using the prototype, ease of use of the app, setup of the prototype, and holder. People seemed excited about the potential applications related to sleep and scent. The majority of people were especially interested in applications that improve their sleep quality (see Figure 10). Seventy-three percent of the participants in the scent condition reported being interested in owning such a device and 46% in control. Ninety percent of the total participants reported that they would use it “daily” or “weekly.” Some randomly picked comments include:

Scent condition: *"The scent made me relaxed when falling asleep, and made the room smell fresher in the morning,” “It was a good experience. However, I managed to knock it down when waking up. But definitely slept better.”, “At first, I fell asleep slowly due to expectation. I woke up several times during the night, but I fell back to sleep more easily and fewer dreams than usual. I felt a bit more strong some times.”, “It was easy to set up and the room smells good now. I couldn't tell much of a difference but certainly a very pleasant experience.”*

Control (water): “*Not much difference from other nights, but I was probably a little too self conscious because of the position of the device. I usually wake up a couple times during the night and when this happened I would immediately look up to see if it was in place.”, “The prototype was very easy to install and use.”, “Worked well. Only challenge if you could call it that was the proximity of the arm of the prototype which I knocked a few times while sleeping with my arm when rolling over. Not a major issue, however.”*

Some comments about their overall experience were: “*I usually have negative, confrontational or violent dreams. Especially if I am too hot. I left my room heater on all night by accident and was sweaty during the night. However, I had either neutral or positive dreams. Also, the scent was very familiar. So when I was confused in the middle of the night whether I was asleep or awake, I smelled the scent and felt comfortable thinking, “Oh I recognize this smell. I must be awake.” Looking back though, I'm not sure if I was smelling the scent in my dreams or in an awake state. At the moment though, it was comforting to think that I know for certain that I am awake because I recognize the smell.”*

Participants found the fragrance pleasant and somewhat strong (as shown in Figure 7). A third of participants noticed the 10 ms burst of scent for only 1 s, 13% for 3 s, 26% for 3 s or more, and 26% for 2 s. For the daytime practice test, participants were fast at refilling the device (with an average time required of ≈28 s) and they found it easy to refill (as shown in Figure 10). Please refer to the auxiliary material for additional information and descriptive statistics.

#### 5.4.1. Burst Duration and Frequency

Forty-six percent of participants would keep the same intensity/frequency settings, 33% would trigger scent less often, 13% more often, and 6% did not mind. Surprisingly, for the control condition (water), 26% of the participants reported “trigger scent less often,” and 13% to trigger “more often.”

#### 5.4.2. A Modular Device vs. Multiple Devices

Participants were also asked to choose between a modular device that can be worn during the day and night (current prototype) or if they would prefer two different devices (one while sleeping, and a different one for the day). Seventy percent of people choose to use only one prototype for its convenience.

## 6. Discussion

Conducting sleep studies remotely and in the wild is challenging. We have shifted the way we test participants due to the COVID-19 pandemic, which also reflected the types of user interfaces we design. There has been an increased interest in studying the coronavirus's sensory impact, especially now that many people are losing their sense of smell. The scientific community has lately been trying to conduct more studies that involve the sense of smell. Still, the technologies used for treatment and therapy are limited, and there is a lack of mobile scent-delivery systems to operate outside of research laboratories.

In our study, participants slept in their homes and most of them were able to use the device successfully. Therefore, this naturalistic setting minimizes the First Night Effect (FNE, Toussaint et al., 1995) and brings new opportunities to potentially use the data of the first experimental night.

It is essential to discuss the challenge of the degree of blindness in olfactory studies. Participants were told that the prototype might trigger a burst of scent in the morning, irrespectively of their assigned condition. Hence, this way even if they could smell the fragrance when waking up in the morning, it would be harder to guess if they were in the control or scent condition. In this study, some participants reported being in an odorless condition, even though they were in the experimental one, suggesting that this approach was useful.

### 6.1. Hygiene and Cross-Contamination

It is also worth considering that, in comparison to audio or visual interfaces, olfactory interfaces require more hygiene and care. Fragrances can be easily cross-contaminated when using a new type of scent in an old capsule. Some scented liquid can remain at the bottom of the piezoelectric, where it was pressed by the cotton filter, which can cause cross-contamination if not cleaned before a new scent is inserted. Small droplets of fragrance might accumulate on the surface of the prototype, especially when the cotton used is soft. Therefore, it is recommended that if the prototype is used for a long duration, the angle between the burst direction and the surface of the device should exceed 90° (e.g., in a horizontal position or upside down like in the study presented). It is also advised to clean the case from time to time. In this study, the piezoelectrics and the top of the capsules were cleaned by rubbing a cotton swab with alcohol and by releasing a couple of bursts of scent with pure alcohol (to clean the microholes of the piezoelectric). The cotton filter was replaced after every use.

### 6.2. Limitations

Further research needs to be conducted, including an objective measurement of physiological or brainwave data from the participants and a sample size larger than 40 users. The rationale behind not using biometric sensors in the current study was to avoid external variables that could have interfered with the olfactory interface ratings. There was a chance that adding sensors to the body or in the bed could negatively affect sleep due to discomfort of wearing a headband. Additionally, the primary interest of this study was on the usability and subjective experience using the olfactory device alone instead of validating the automatic sleep stage algorithm compared to Polysomnography. In future studies—and now that the olfactory device alone has been studied—the device can be compared to a traditional olfactometer and in a sleep laboratory. One last limitation worthy of mentioning is the complexities and artifacts that the EEG signal possesses while recording, especially in a mobile setting or unsupervised at home. Therefore, to avoid noise artifacts (especially those generated from the eye movements), we recommend choosing the Tp10 electrode for classification instead of the frontal electrodes (Af8, Af7).

### 6.3. Ethical Implications

Sleep interfaces that track our physiological signals, such as heart rate or brain waves, sleep patterns, and even potentially dream content, should be regulated to protect the individual's privacy and always to prioritize the users' wellbeing. In this research, some of these challenges were addressed by developing on-device machine learning that can run on the user's phone and using BLE instead of sending the data via WiFi to a server; hence the data is locally stored on the user's phone. The EEG device can monitor spontaneous activity from the brain to infer their sleep stages that are only displayed in their smartphone, and the user always has the control over the olfactory feedback provided at night by presetting the intensity and frequency on the app. However, there are still many challenges that need to be addressed and recognized when creating or using sleep UIs. It is not enough simply to note the existence of privacy challenges, and ethical codes for researchers, the potential misuses of these technologies should be spelled out to minimize the unexpected, adverse outcomes and maximize their positive impact. Sleep UIs can provide unique positive outcomes that otherwise might not be possible with regular UIs used during wakefulness. For example, researchers like Hu et al. (2015) and Arzi et al. (2014) are investigating and have shown promising results to address unconscious behaviors such as cognitive biases, phobias, or addictions.

### 6.4. Future Directions

A future research direction is to conduct a feasibility study in sleep laboratories and a clinical setting. We have already started running preliminary tests in a sleep laboratory with a high-density EEG (see Supplementary Material for details on the preliminary results). The results suggest that there is potential to conduct further studies combined with wearable EEGs and automatic sleep scoring (like the one presented in this article) as an alternative to high-density EEGs and manual sleep scoring and scent release. These systems could also be combined with wristbands to deliver heart rate and electrodermal activity on sleep-related events to the scent-delivery prototype. The hope is that the research presented in this article is a step toward making sleep experiments more accessible to everyone, including those that might not have the resources to build or access sleep laboratory equipment and to foster new ways to collaborate across disciplines. There are numerous applications to design Olfactory Interfaces based on proxemics and how active the interaction for Sleep UIs is. For example, scent can be released in a mobile and discrete manner during the day in indoor and outdoor environments. The same odor can be reactivated during specific sleep stages at night, strengthening the efficacy of such interventions. Some examples include using scent for targeted memory reactivation during sleep, reducing certain types of addictions (such as smoking behavior), scent-induced lucid dreaming as well as targeted memory reactivation for wellbeing applications (such as using scent during a meditative experience and releasing it while sleeping to reactivate that emotional state). There are many other futuristic, science fiction-inspired, and intriguing applications, such as storytelling through dreams and dream communication. However, the most beneficial and realistic applications for the design of technologies for the home environment in the next decade will be wellbeing-related. Improve sleep quality, reduce insomnia, nightmares, or maladaptive memories, and ease sleep apnea, snoring, jaw clenching, and counteract sleep disorders caused by stress and anxiety. Additionally, it would be interesting to investigate how the framework for Olfactory Proxemics defined in this article might apply to co-located users who need a remote synchronization of scent over distance. For example, remote communication, social media, telecommunication, remote VR applications, and co-located sleepers.

## 7. Conclusions

This article describes the development, study, and framework for Olfactory Interfaces and sleep. We describe these explorations in the context of their use in developing a modular, closed-loop sleep olfactory interface and a preliminary sleep experiment with 40 participants. The study results indicate that participants were satisfied with the prototype, the holder, and the smartphone application. They found the app easy to use, the device easy to refill, and the holder easy to set up. Compared to participants in the control condition (using Ezzence with water), those in the scent condition significantly improved their subjective sleep quality with respect to their regular sleep. More than three-quarters of the participants in the scent condition (using Ezzence with lavender, clary sage, and copaiba oil) did not report awakenings caused by the device. Furthermore, a significant number of participants using Ezzence with scent reported better mood the following morning than on their regular basis and reported falling asleep faster. Besides the study and development of this device, this article presents a framework on Sleep UIs' (user interfaces for sleep environments) and “Olfactory Proxemics” (scent interactions based on the distance between the origin of the scent-delivery device and the user).

This research aimed to shed light on using scent to improve wellbeing in-home sleep environments. We hope that the detailed reporting of the considerations when developing these interfaces and wearable scent-delivery systems will prove helpful to researchers from many fields, engineers, designers, and scientists alike.

## Data Availability Statement

The original contributions presented in the study are included in the article/Supplementary Material, further inquiries can be directed to the corresponding author/s.

## Ethics Statement

The studies involving human participants were reviewed and approved by the Committee on the Use of Humans as Experimental Subjects (COUHES) at the Massachusetts Institute of Technology (MIT). The patients/participants provided their written informed consent to participate in this study. Written informed consent was obtained from the individual(s) for the publication of any potentially identifiable images or data included in this article.

## Author Contributions

JA and PM designed the experiment. JA and MD developed Ezzence and carried out the experiments for all participants. JA analyzed the data and wrote the article. PM provided feedback about the data and article. MD provided help with figures and the mechanical design part of the article. All authors reviewed the manuscript. All authors contributed to the article and approved the submitted version.

## Funding

This work was supported by the MIT Media Lab.

## Conflict of Interest

The authors declare that the research was conducted in the absence of any commercial or financial relationships that could be construed as a potential conflict of interest.

## Publisher's Note

All claims expressed in this article are solely those of the authors and do not necessarily represent those of their affiliated organizations, or those of the publisher, the editors and the reviewers. Any product that may be evaluated in this article, or claim that may be made by its manufacturer, is not guaranteed or endorsed by the publisher.

## Footnotes

^1^ https://www.youtube.com/watch?v=0MZ9yu3d5bM

^2^ https://en.wikipedia.org/wiki/Smell-O-Vision
