# Supplementary material for: Development and Study of Ezzence: A Modular Scent Wearable to Improve Wellbeing in Home Sleep Environments
Source: Front Psychol. 2022 Mar 17;13:791768. doi: 10.3389/fpsyg.2022.791768 (PMC8970317; doi:10.3389/fpsyg.2022.791768)
Supplement: Supplementary file 1 [file Data_Sheet_1.PDF]

Supplementary Material

1 SUPPLEMENTARY MATERIAL

The analysis, Python code and data can be downloaded as a complement to the material of this paper. We created a Jupyter notebook that is publicly available on **GitHub**[1].

2 SUPPLEMENTARY STUDY

Figure S1 shows the brain topographic map during slow-wave sleep when using the device. An odor exposure during ten milliseconds successfully increased slow-wave activity (SWA). These preliminary findings are promising since SWA plays a crucial role in memory consolidation. Thus, using scent might be a promising intervention for those suffering from memory loss, cognitive impairment and as a potential way to improve memory problems resulting from traumatic experiences. Therefore, identifying techniques to enhance SWA that are feasible for long-term use outside the sleep laboratory can be very beneficial, especially for clinical populations. In this preliminary test, sleep was scored by looking in real-time the brain activity pattern on a computer screen and releasing a burst of odor by manually pressing the button in the smartphone app when the user was entering slow-wave sleep. This method is usually conducted using a large olfactometer connected to a nasal mask that is manually activated through a computer. With our system, scent can be triggered manually or automatically in an autonomous manner.

[1] https://github.com/jdthamores/ezzence-data-analysis-study-usability

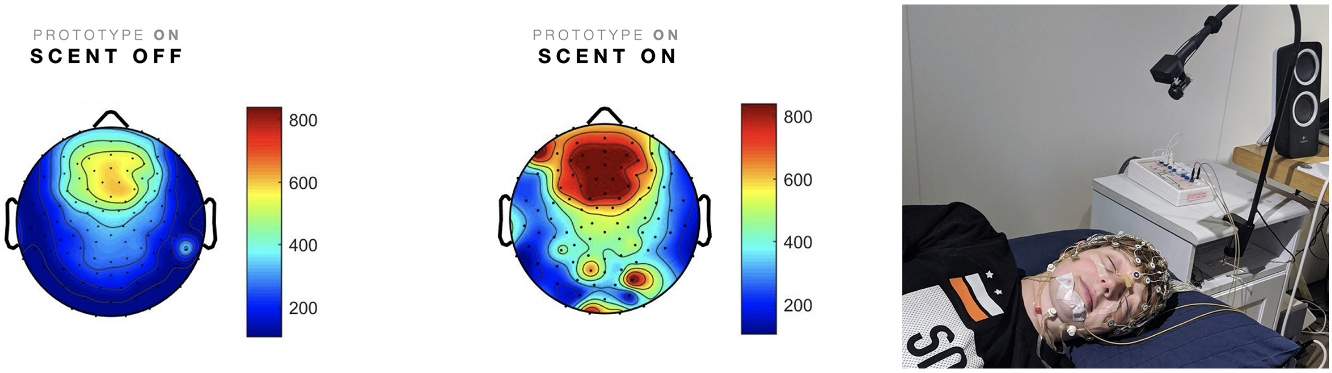

Figure S1. On the right, a participant is wearing a 128 electrode EEG while the prototype is "on". The two images on the left represent the mean EEG power in (microvolts) across all slow-wave activity episodes in one participant while the prototype was on. On the left, while the prototype was not releasing scent, and on the right, during the 10 ms burst of scent.

3 SUPPLEMENTARY DATA

The study design is relatively simple: only two groups (scent versus control), thus to test if the differences between the scent and the control group's means were statistically significant, we first examined if the assumptions to perform a two-sample t-test were met. We checked the normality of the data graphically, through a Shapiro-Wilk Test, analyzed the skewness and kurtosis and ran a Levene test to analyze the variances of the data. Based on these results and the data distribution, we ran a non-parametric Wilcoxon (Mann–Whitney) test. However, the parametric t-test has been shown to be robust against non-normality, and some argue that there is nearly no need to use Wilcoxon (Mann–Whitney) non-parametric test (**?**). Therefore, for completeness, we ran both tests. Both tests show a statistically significant difference between the means of the two groups (scent versus control) in the same cases (sleep quality, mood, sleep latency, scent awareness). The supplementary material provides more details on the analysis, the Python code, and data.

3.1 Tables

Table S1. Satisfaction and ease of using the prototype, holder and app. Mean and standard error mean are reported.

	Descriptive Statistics	
**User Experience**	Mean	*SEM*
Satisfaction using the prototype	6.13	0.15
Ease of refill	6.06	0.26
Form factor (physical design of the prototype without the sleep holder)	5.83	0.19
Design prototype + holder	5.8	0.23
Ease of use of app	5.8	0.24
Ease of adjusting the prototype	5.6	0.26

Likert Scale Values: 7 is "very positive" and a 1 is "very negative". A neutral response is a 4.

Table S2. Descriptive Statistics. Participants wore the prototype during the daytime olfactory test and rated how strong and pleasant was the fragrance.

	Daytime Olfactory Test	
**Odor Perception**	Mean	*SEM*
Strength	4.9	0.21
Pleasantness	5.8	0.19

Likert Scale Values: 7 is "very strong" and a 1 is "very light". A neutral response is a 4.
For pleasantness, a 7 is "very pleasant" and a 1 is "very unpleasant".

Table S3. Relative changes of sleep quality for scent and control condition with respect to a typical night. Participants were asked to rank their sleep quality on the night of the study using a 7-point Likert Scale (1= Very bad, 7 = very good), as well as the following question: *What's your sleep quality on a typical night? (1 = Very bad, 7 = Very good).* We calculated the difference between a typical night and the night of the study and set as 100% (best) their original sleep. The table summarizes the descriptive and inferential statistics, including Shapiro-Wilk test for normality and the Levene test for variances. Given these results and given the robustness of the t-test with not normal data, we conducted both parametric (t-test for the independent samples) and non-parametric tests (Mann–Whitney U test / Wilcoxon rank-sum test).

**Sleep Quality**	*M*	*SD*	*SEM*	Median	Skew.	Kurt.	Wilk	Levene	*t-test (p)*	*df*	*t*	*U-test (p)*	*U*
Scent	19.29	22.0	5.88	10.0	0.459	-1.748	0.002	0.1998	**0.0037**	29	2.89	**0.0091**	157
Control	-2.24	17.95	4.63	0.0	-0.156	-0.837	0.229						

Relative sleep quality: improvement (%) with respect to baseline (typical night).
Descriptive and inferential statistics (one tail independent t-test and Mann–Whitney U test reported). Participants in the scent group reported a significant improvement in their sleep quality compared to the control group.

Table S4. Perceived Sleep & Mood: Relative changes of perceived mood the following morning, perceived depth of sleep, perceived rest, content of positive dreams and time to fall asleep for scent and control condition with respect to a typical night. Participants answered their questions using a 3-point Likert Scale (+1 when mood/deep sleep, etc were improved, -1 when they decreased compared to a typical night, and 0 if it was like an average sleep). For plotting and analyzing the data, the results have been set as 100% when improved (equivalent of +1), -100% when decreased (-1), and 0 if there was no change.

	Scent				*Control*							
**Self-reported sleep & mood**	*M*	Median	*SEM*	*SD*	*M*	Median	*SEM*	*SD*	*t-test (p)*	*t*	*U-test (p)*	*U*
Mood	64.29	100.0	13.29	49.72	26.67	0.0	15.33	59.36	**0.03818**	1.843	**0.04369**	140
Deep Sleep	42.86	0.0	13.73	51.36	33.33	0.0	12.6	48.8	0.30637	0.5121	0.31099	115
Perceived Rest	42.86	0.0	13.73	51.36	26.67	0.0	15.33	59.36	0.22025	0.7829	0.24740	119
Pos. Dreams	21.43	0.0	15.47	57.89	6.67	0.0	15.33	59.36	0.25202	0.6772	0.25785	118
Sleep Latency	42.86	50.0	17.27	64.62	-20.0	0.0	17.46	67.61	**0.00827**	2.556	**0.01017**	154.2

Relative improvement with respect to a typical night. (%). Descriptive and inferential statistics (one tail independent t-test and Mann–Whitney U test reported). Participants in the scent group reported a significant improvement in their mood and reduced time to fall asleep compared to the control group.

Table S5. Prototype, scent and sound awareness during sleep for scent and control condition. Participants answered using a 7-point Likert Scale (1= very aware, 7 = very unaware). The table summarizes the descriptive and inferential statistics, including t-test for the independent samples and Mann–Whitney U test / Wilcoxon rank-sum test.

	Scent				*Control*							
**Awareness & Awakenings**	*M*	Median	*SEM*	*SD*	*M*	Median	*SEM*	*SD*	*t-test (p)*	*t*	*U-test (p)*	*U*
Scent Awareness	4.36	5.0	0.48	1.78	6.13	7.0	0.43	1.68	**0.005**	2.76	**0.001**	39.5
Prototype Awareness	5.29	6.0	0.44	1.64	5.07	5.0	0.5	1.94	0.373	0.326	0.491	106
Sound Awareness	6.36	7.0	0.36	1.34	6.13	7.0	0.43	1.68	0.348	0.394	0.299	115

Descriptive and inferential statistics (one tail independent t-test and Mann–Whitney U test reported). There was a significant difference between the scent and control group for awareness of the smell during sleep.
